# Supplementary material for: A Sensitivity-Optimized Flexible Capacitive Pressure Sensor with Cylindrical Ladder Microstructural Dielectric Layers
Source: Sensors (Basel). 2023 Apr 27;23(9):4323. doi: 10.3390/s23094323 (PMC10181647; doi:10.3390/s23094323)
Supplement: Supplementary file 1 [file sensors-23-04323-s001.zip › sensors-2341174-supplementary.pdf]

Figure S1 displays the uniform size of 9 microstructures. The base length and height of these nine microstructures are the same. Here we take the cylindrical ladder microstructure as a representative, please refer to Figure S1, the length and width of the 9 microstructures are all the way 610 μm, the height is all the way 250 μm.

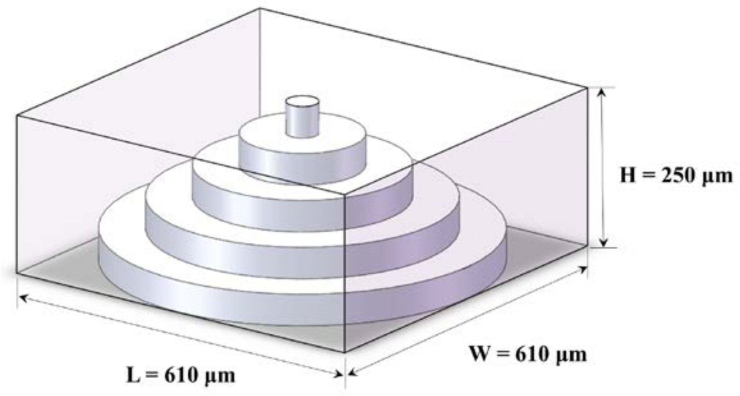

Figure S1. Description of the size of the microstructure (cylindrical ladder microstructure is taken as an example)

Figure S2 displays the sensitivity obtained by the stretching machine test of sensor prototype is basically consistent with the simulation result. Please note that when referring to Figure S2, because the sensitivity obtained in the experiment is measured from the sensor prototype, and for the simulation we simulate the sensitivity of a single microstructure, so there are some differences between the experimental data and the simulated data, but the trend of change is consistent.

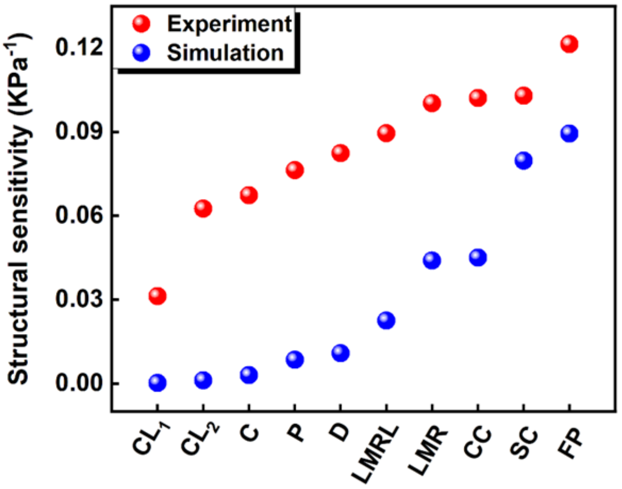

Figure S2. Simulation and experimental results of the sensitivity with different microstructures.

Figure S3 displays scanning electron microscope images of molds exhibiting nine surface microstructures. The largest size of these microstructures should be 610 μm, where this value represents the width of the long micro ridge ladder and long micro-ridges structures. The pitch between the microstructures is 1220 μm. However, due to the shrinkage of the resin after light curing, the size will be reduced by 97–98%. The surface area of the mold exhibiting the microstructures measures 1.25 cm × 1.25 cm, with a total of 10 × 10 microstructures present. In the case of the long micro ridge ladder and long micro-ridge structures, they are evenly distributed, with 10 instances of each present on the mold's surface. These SEM pictures do not provide an overview of the entire mold.

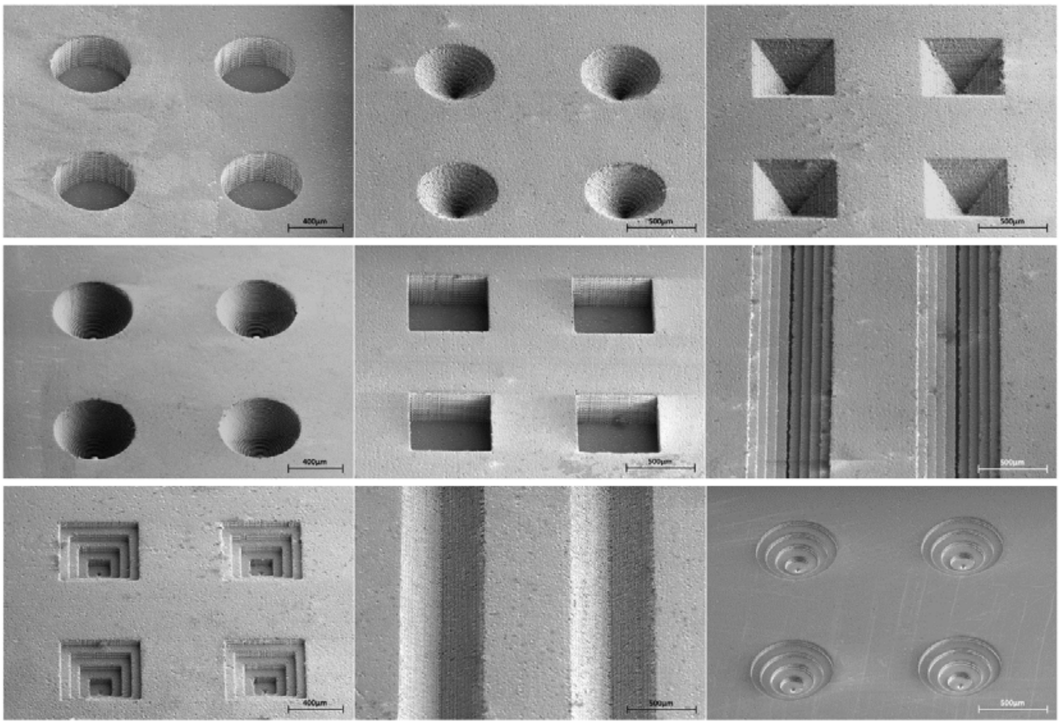

Figure S3. Scanning electron microscope images of molds exhibiting nine surface microstructures.

Figure S4 displays the SEM image of the cylindrical ladder microstructure's surface, which reveals that it is not a smooth transition. This can be attributed to the 3D printing method utilized during the mold's fabrication process. Specifically, the printing layers had a controlled thickness of 10 μm, resulting in an interlayer structure that cannot be removed. Therefore, the layer-by-layer printing technique led to the formation of an uneven surface topography on the mold's microstructures.

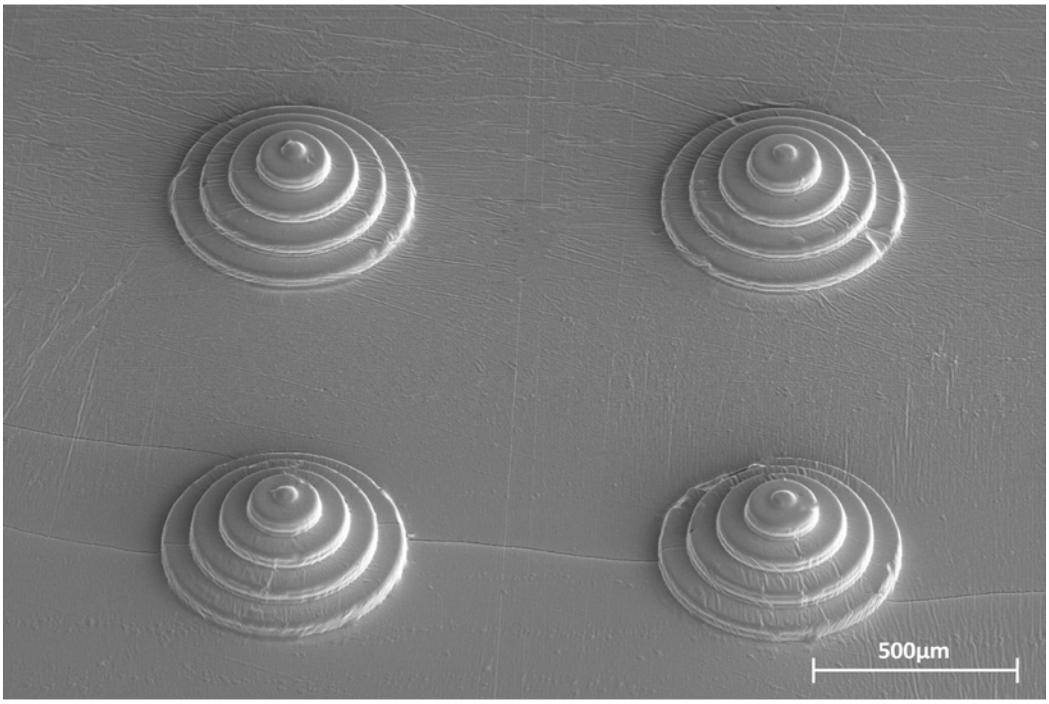

Figure S4. Scanning electron microscope images of surface morphology of cylindrical ladder microstructures

Figure S5 displays the pressure-capacitance change rate of the sensor under different bending radii. By sticking the flexible capacitive pressure sensor on molds with different bending radii for pressure measurement, we found that under different bending radii conditions(30 mm, 50 mm, 70 mm and flat surface), the developed

flexible capacitive pressure sensor has a stable output under different pressures, and the capacitance change rate of the output signal does not change due to the change of the bending radius.

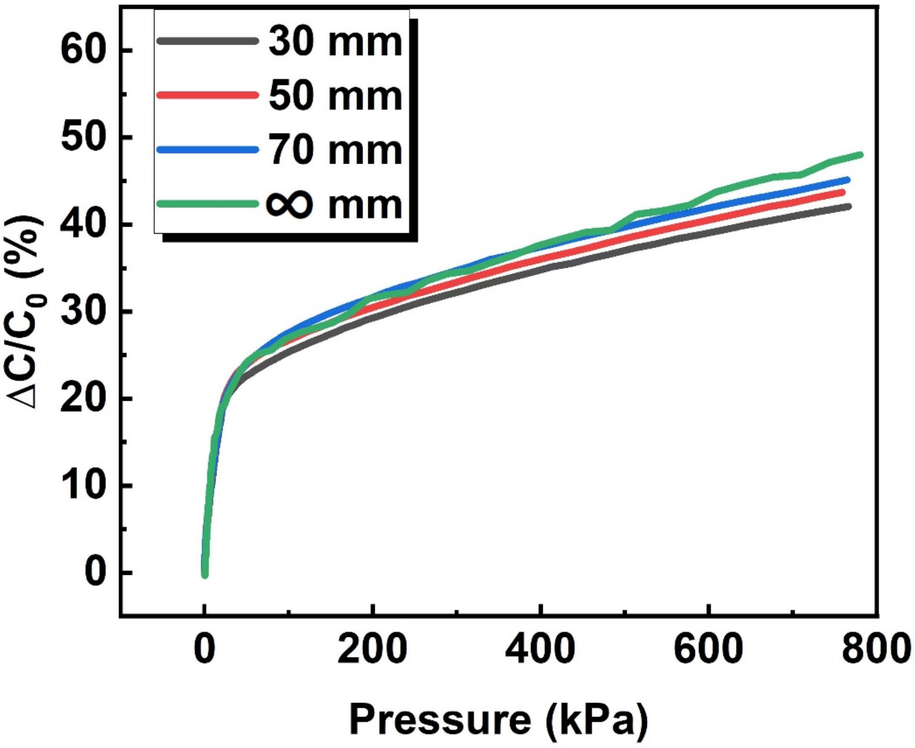

Figure S5. The pressure-capacitance change rate of the sensor under different bending radii.
